# Intensity Zones and Intensity Thresholds Used to Quantify External Load in Competitive Basketball: A Systematic Review

**DOI:** 10.1007/s40279-024-02058-5

**Published:** 2024-06-18

**Authors:** Matthew C. Tuttle, Cody J. Power, Vincent J. Dalbo, Aaron T. Scanlan

**Affiliations:** School of Health, Medical and Applied Sciences, Central Queensland University, Rockhampton, QLD Australia

## Abstract

**Background:**

Despite widespread use of intensity zones to quantify external load variables in basketball research, the consistency in identifying zones and accompanying intensity thresholds using predominant monitoring approaches in training and games remains unclear.

**Objectives:**

The purpose of this work was to examine the external load intensity zones and thresholds adopted across basketball studies using video-based time-motion analysis (TMA), microsensors, and local positioning systems (LPS).

**Methods:**

PubMed, MEDLINE, and SPORTDiscus databases were searched from inception until 31 January 2023 for studies using intensity zones to quantify external load during basketball training sessions or games. Studies were excluded if they examined players participating in recreational or wheelchair basketball, were reviews or meta-analyses, or utilized monitoring approaches other than video-based TMA, microsensors, or LPS.

**Results:**

Following screening, 86 studies were included. Video-based TMA studies consistently classified jogging, running, sprinting, and jumping as intensity zones, but demonstrated considerable variation in classifying low-intensity (standing and walking) and basketball-specific activities. Microsensor studies mostly utilized a single, and rather consistent, threshold to identify only high-intensity activities (> 3.5 m·s^−2^ for accelerations, decelerations, and changes-in-direction or > 40 cm for jumps), not separately quantifying lower intensity zones. Similarly, LPS studies predominantly quantified only high-intensity activities in a relatively consistent manner for speed (> 18.0 m·s^−1^) and acceleration/deceleration zones (> 2.0 m·s^−2^); however, the thresholds adopted for various intensity zones differed greatly to those used in TMA and microsensor research.

**Conclusions:**

Notable inconsistencies were mostly evident for low-intensity activities, basketball-specific activities, and between the different monitoring approaches. Accordingly, we recommend further research to inform the development of consensus guidelines outlining suitable approaches when setting external load intensity zones and accompanying thresholds in research and practice.

## Key Points

**Table:** 

This systematic review identified inconsistencies in the intensity zones and intensity thresholds adopted within and between popular monitoring technologies (i.e., video-based TMA, microsensors, and LPS) in the basketball literature.
Across the existing research using different technologies to monitor external load using intensity zones in basketball, studies have frequently quantified only high-intensity activities, relied on manufacturer settings to set intensity zone thresholds, and sparingly included relative intensity zones.
Further research encompassing expert input alongside validation work is needed to identify best-practice approaches that guide end-users in setting external load intensity zones and intensity thresholds in research and practice.

## Introduction

Basketball is a court-based team sport that requires players to perform intermittent, high-intensity activity including sprinting, cutting, shuffling, and jumping during training and games [1]. These demands can impose high external loads on players, whereby the external load represents the physical stimuli experienced during training and games [2, 3]. In turn, quantifying external load variables can be useful for basketball coaches to confirm they are delivering the desired stimuli to players to optimally prepare them for competition and safeguard them against excessive physical stress [4]. In this regard, external load variables have primarily been quantified in basketball players using video-based time-motion analysis (TMA), microsensors, and local positioning systems (LPS).

Video-based TMA utilizes video footage of training sessions and games to track player movements and identify the type and intensity of movement to obtain several external load variables such as movement frequencies, durations, distances, and speeds [5]. Historically, manual player tracking involving subjective identification of movement types via visual recognition has been adopted when using video-based TMA systems in basketball. However, this approach is labor- and time-intensive, restricting the ability to provide real-time feedback to coaches in practice [2, 6]. In turn, technological advancements have allowed for more efficient measurement of the same external load variables using video-based TMA systems, with various software platforms now possessing automated player tracking capabilities. Automated tracking in video-based TMA systems has removed the subjectivity accompanying visual interpretation of footage inherent in manual tracking systems, providing variables with quicker turnaround times for coaches to use [7]. Likewise, technological advancements underpin the increased use of microsensors and LPS to measure external load variables in basketball players over the past decade. In this regard, microsensors are small devices worn by players during training sessions and games, which typically contain accelerometers, gyroscopes, and magnetometers to quantify player movements in multiple directions [8]. In turn, microsensors provide efficient data collection and processing, can be easily transported for use in various locations, and provide several variables including accelerations, decelerations, changes-in-direction, jumps, and PlayerLoad [9]. In this regard, PlayerLoad is a composite variable that combines the instantaneous rates of change in acceleration across the three movement axes via the following formula: √((Ac1_*n*_ − Ac1_*n*–1_)^2^ + (Ac2_*n*_ − Ac2_*n*–1_)^2^ + (Ac3_*n*_ − Ac3_*n*–1_)^2^) [10]. Moreover, LPS utilize radio frequency, Bluetooth, or ultra-wideband technologies to track player position on the court via worn sensors [11]. LPS allow for efficient data collection and processing, while providing distance-, speed-, and acceleration-derived variables [12].

Despite the various technologies and processes underpinning TMA, microsensors, and LPS, each provides distinct and overlapping variables indicative of exercise intensity. Intensity is generally considered as the physical work completed per unit of time in regard to external load monitoring [13]. Intensity is an important component of external load given it is often manipulated by coaches during periodized training plans across different seasonal cycles in basketball players [14–17], while also being a key contributing factor in obtaining desired physiological adaptations in response to prescribed training [17, 18]. Furthermore, quantification of the external load intensities encountered during training and games is essential to understanding the specificity of prescribed drills in relation to competition demands. In turn, external load intensity has been frequently quantified with intensity zones predicated on distinct thresholds to delineate these zones for specific variables (e.g., speed, acceleration, deceleration) in basketball research. However, the extent of consistency in methodologies to demarcate intensity zones for external load variables measured within and between popular monitoring approaches remains unclear in the basketball literature. If inconsistent approaches to define intensity zones and thresholds have been applied in basketball research, such as that identified systematically across various field-based team sports [19–22], meaningful comparisons across studies and a definitive consensus regarding the external load intensities experienced by basketball players cannot be effectively elucidated. Therefore, this systematic review aims to determine whether variation exists in approaches to classify external load intensity zones and thresholds for each predominant monitoring technology (i.e., video-based TMA, microsensors, and LPS) in basketball research.

## Methods

### Search Strategy

Studies were identified via PubMed, MEDLINE, and SPORTDiscus databases using the search terms presented in Table 1. Studies published online or in print from database inception until 31 January 2023 were considered for inclusion.

**Table 1:** Search string used to locate relevant studies

(zones OR bands OR speed OR intensity OR acceleration OR deceleration OR workload OR training load OR demands OR load OR external OR work OR performance) AND (time motion OR time motion analysis OR TMA OR microsensor OR accelerometers OR local positioning system OR LPS OR optical tracking OR optical sensors OR camera OR monitor OR ultra-wide band OR inertial) AND basketball	

### Selection Criteria

Screening of studies retrieved from database searches was undertaken in accordance with the Preferred Reporting Items for Systematic Reviews and Meta-Analyses (PRISMA) guidelines [23]. Studies considered for inclusion were original peer-reviewed research, published in English, and reporting external load variables identified using intensity zones in competitive basketball players with video-based TMA, microsensors, or LPS. No restrictions were made on the basis of the age or sex of the players examined, study design adopted, or the context of data collection (i.e., games versus training). Exclusion criteria included studies examining players participating in recreational basketball or wheelchair basketball due to varied demands encountered [24], reviews or meta-analyses, and studies using monitoring approaches other than video-based TMA, microsensors, and LPS to measure external load variables. In this regard, while research has utilized global positioning system (GPS) technology to quantify external load variables in basketball players [25], GPS technologies are not readily implemented by basketball coaching staff in practice due to the signal interference encountered when indoors [26]. Likewise, optical tracking systems have been sparsely used to quantify external load variables in basketball players across the literature [4, 27, 28], with their uptake also currently limited in practice [4]. Some of the reasons for the limited uptake of optical tracking systems in research and practice may be the significant costs associated with their use, lengthy data processing time delaying feedback to coaches, and permanence of camera placement restricting data to games at home venues rather than also in training and away venues [29]. For these reasons, studies utilizing GPS or optical tracking systems to quantify external load variables with associated intensity zones were excluded from this review.

Following the removal of duplicates, the titles and abstracts of identified studies in the search were reviewed independently by two authors (M.C.T. and C.J.P.). Any disagreements between the two authors regarding study inclusion after review of the title and abstract were discussed, and if no consensus was reached, a third author (A.T.S.) provided a consensus decision on inclusion. Following the review of titles and abstracts, full-text versions of relevant studies were obtained and screened by two authors (M.C.T. and C.J.P.) to further determine their eligibility for inclusion. Any disagreements between the two authors regarding study inclusion after review of the full-text versions were discussed, and if no consensus was reached, a third author (A.T.S.) established a consensus decision on inclusion.

### Data Extraction and Analysis

Data were extracted from each study by the lead author (M.C.T.), with co-authors reviewing extracted data for accuracy and completeness. The following data were extracted from each study: number of players recruited, mean ± standard deviation (SD) player age, player sex, playing level at which players competed, monitoring approach used for measuring external load variables, hardware and software used to quantify external load variables, number of intensity zones used (e.g., delineating speed into walking, jogging, running, and sprinting zones would equate to four intensity zones), and intensity zone thresholds applied [e.g., speed thresholds applied for jogging may be between 1 m·s^−1^ (lower threshold) and 3 m·s^−1^ (higher threshold)]. Intensity zone and threshold data for all external load variables reported in the included studies were extracted and analyzed. Playing level was categorized from lowest to highest as club, high school, college/university, representative (trained athletes selected into a representative team), semi-professional (some players are full-time and/or contracted athletes), or professional (all players are full-time, contracted athletes) [30]. When at least two studies reported thresholds for the same intensity zone using the same technology (i.e., video-based TMA, microsensors, or LPS), the mean ± SD thresholds were calculated across those studies. Of note, given most studies (5 out of 6, 83%) implemented numeric intensity zone thresholds measured in m·s^−1^ when using video-based TMA, the single study implementing thresholds in km × h^−1^ [31] was manually converted to m·s^−1^ by multiplying threshold values by 5/18 for consistency in reporting. Likewise, given that deceleration intensity zone thresholds were incorrectly reported as positive values instead of negative values in some studies using microsensors [15, 32–38] and LPS [39–44], all deceleration intensity zone thresholds were treated as negative values when calculating mean ± SD thresholds across studies. If a study included multiple player samples with different intensity zone thresholds applied for each sample (e.g., separate player samples competing at different playing levels with different intensity zone thresholds adopted for each playing level) [45], each set of intensity zones was included separately when calculating mean ± SD thresholds across studies.

### Assessment of Methodological Quality

A modified version of the Downs and Black checklist was utilized to evaluate methodological quality of the included studies [46] (Table 2). The modified Downs and Black checklist was chosen as its validity has been established for evaluating experimental [47] and observational studies [48], and it has been adopted previously in other reviews focused on load monitoring in basketball [30, 49]. For this review, 11 of the 27 items from the original checklist were used, which has been previously used in other reviews examining external load in basketball [30, 46, 50]. The quality of each included study was assessed independently by two authors (M.C.T. and C.J.P.) with each item scored as “1” (yes) or “0” (no/unable to determine). The scores for each of the 11 items were then summed to provide the total quality score. Any disagreement in the outcome of the quality appraisal for individual studies was reviewed by a third author (A.T.S.) for a consensus decision.

**Table 2:** Modified Downs and Black checklist used to assess methodological quality and risk of bias in the included studies

Number	Question
*Reporting*	
1	Is the hypothesis/aim/objective of the study clearly described?
2	Are the main outcomes to be measured clearly described in the “Introduction” or “Methods” section?
3	Are the characteristics of the patients included in the study clearly described?
4	Are the main findings of the study clearly described?
5	Does the study provide estimates of the random variability in the data for the main outcomes?
6	Have actual probability values been reported (e.g., 0.035 rather than < 0.05) for the main outcomes except where the probability value is less than 0.001?
*External validity*	
7	Were the subjects asked to participate in the study representative of the entire population from which they were recruited?
8	Were those subjects who were prepared to participate representative of the entire population from which they were recruited?
*Internal validity*	
9	If any of the results of the study were based on “data dredging,” was this made clear?
10	Were the statistical tests used to assess the main outcomes appropriate?
11	Were the main outcome measures accurate (valid and reliable)?

## Results

### Study Selection and Methodological Quality

**Fig. 1:**
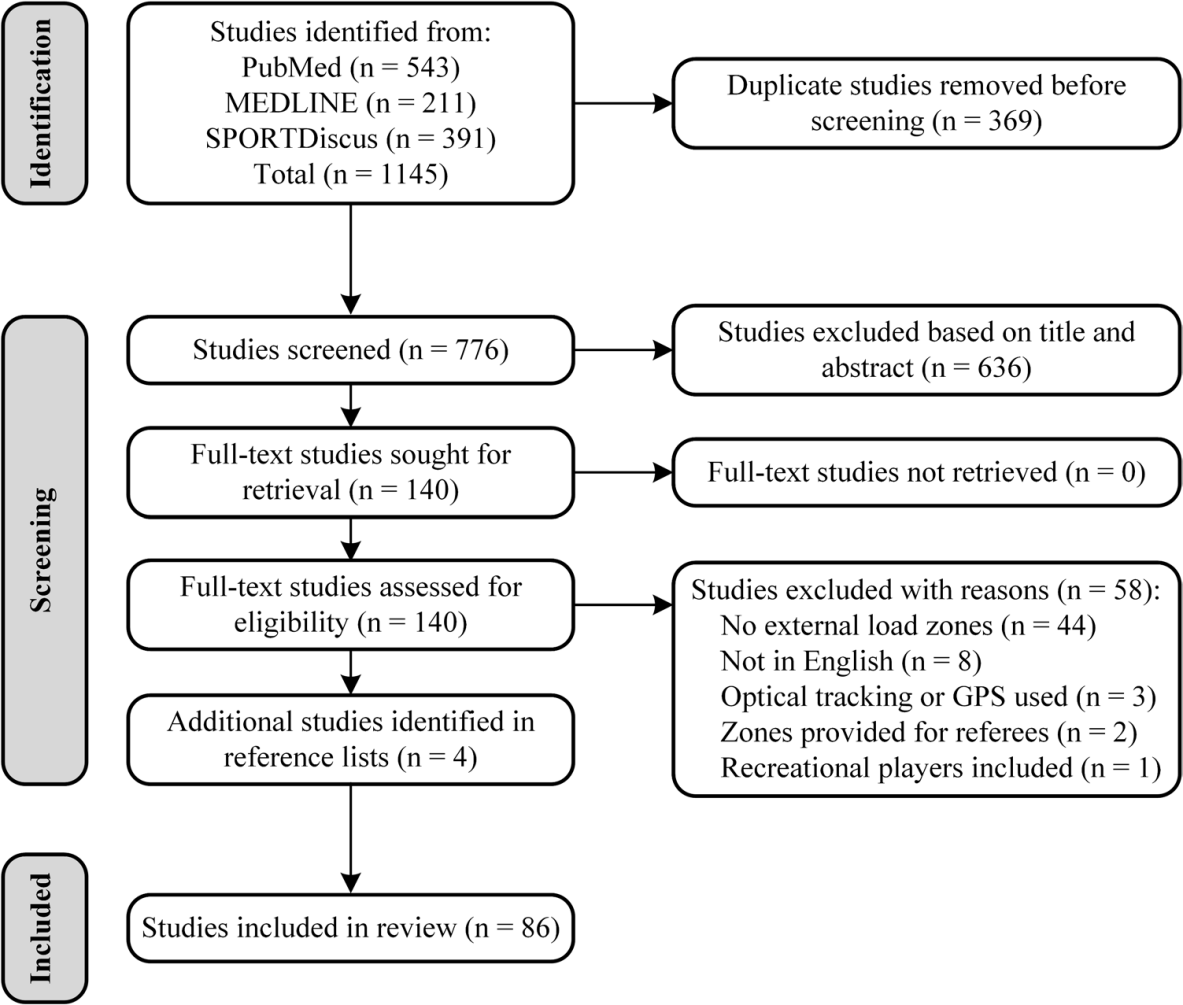
Preferred Reporting Items for Systematic Reviews and Meta-Analyses (PRISMA) flow diagram showing the search results for this review

The results of the search process are presented in Fig. 1. The initial search across databases retrieved 1145 studies, with 369 studies identified as duplicates and a further 636 studies excluded on the basis of their title or abstract. Accordingly, 140 full-text studies were screened, with 58 of these studies being excluded, leaving 82 studies for further analysis. An additional four studies were retrieved from the reference lists of the 82 included studies, resulting in 86 studies being included in our analysis. Methodological quality and risk of bias scores ranged from 7 to 11 out of 11 (mean ± SD: 9.0 ± 0.9), with no studies being excluded on the basis of quality (Table 3).

**Table 3:** The methodological quality scores for included studies

Study	Downs and Black checklist question number											Total
	1	2	3	4	5	6	7	8	9	10	11	
Abdelkrim et al. [105]	1	1	1	1	1	1	0	0	1	1	1	9
Abdelkrim et al. [31]	1	1	1	1	1	0	0	0	1	1	1	8
Abdelkrim et al. [5]	1	1	1	1	1	0	0	0	1	1	1	8
Abdelkrim et al. [106]	1	1	1	1	1	0	1	1	1	1	1	10
Alonso Perez-Chao et al. [39]	1	1	1	1	1	1	0	0	1	1	1	9
Alonso Perez-Chao et al. [40]	1	1	1	1	0	1	1	1	1	1	1	10
Aoki et al. [14]	1	1	1	1	1	0	1	0	1	1	1	9
Arede et al. [86]	1	1	1	1	1	0	0	0	1	1	1	8
Arede et al. [90]	1	1	1	1	1	1	0	0	1	1	1	9
Ball et al. [57]	1	1	1	1	1	1	0	0	1	1	1	9
Bishop et al. [51]	1	1	1	1	1	0	0	0	1	1	1	8
Bredt et al. [64]	1	1	1	1	1	1	1	1	1	1	1	11
Bredt et al. [65]	1	1	1	1	1	1	1	1	1	1	1	11
Castillo et al. [78]	1	1	1	1	1	1	1	1	1	1	1	11
Castillo et al. [87]	1	1	1	1	1	1	0	0	1	1	1	9
Conte et al. [107]	1	1	1	1	1	0	0	1	1	1	1	9
Delextrat et al. [108]	1	1	1	1	1	1	0	0	1	1	1	9
Delextrat et al. [109]	1	1	1	1	1	0	0	0	1	1	1	8
Ferioli et al. [110]	1	1	1	1	1	1	1	0	1	1	1	10
Ferioli et al. [111]	1	1	1	1	1	1	1	0	1	1	1	10
Ferioli et al. [112]	1	1	1	1	1	1	0	0	1	1	1	9
Ferioli et al. [138]	1	1	1	1	1	0	1	1	1	1	1	10
Fox et al. [58]	1	1	1	1	1	1	0	0	1	1	1	9
Fox et al. [33]	1	1	1	1	1	0	0	0	1	1	1	8
Fox et al. [34]	1	1	1	1	1	1	0	0	1	1	1	9
Fox et al. [32]	1	1	1	1	1	1	0	0	1	1	1	9
Fox et al. [59]	1	1	1	1	1	1	0	0	1	1	1	9
Garcia et al. [91]	1	1	1	1	1	0	1	1	1	1	1	10
Garcia et al. [92]	1	1	1	1	1	0	1	1	1	1	1	10
Garcia et al. [41]	1	1	1	1	1	0	0	0	1	1	1	8
Garcia et al. [42]	1	1	1	1	1	0	0	0	1	1	1	8
Garcia et al. [43]	1	1	1	1	1	0	0	0	1	1	1	8
Garcia et al. [93]	1	1	1	1	1	0	0	0	1	1	1	8
Heishman et al. [139]	1	1	1	1	1	1	1	1	1	1	1	11
Hulka et al. [52]	1	1	1	1	1	1	0	0	1	1	1	9
Ibanez et al. [45]	1	1	1	1	1	1	1	0	1	1	1	10
Klusemann et al. [113]	1	1	1	1	1	0	0	0	1	1	1	8
Koyama et al. [66]	1	1	1	1	1	0	0	0	1	1	1	8
Koyama et al. [67]	1	1	1	1	1	1	0	0	1	1	1	9
Leicht et al. [69]	1	1	1	1	1	0	0	0	1	1	1	8
Matthew et al. [114]	1	0	1	1	1	0	0	0	1	1	1	7
McInnes et al. [104]	1	1	1	1	0	0	0	0	1	1	1	7
Nagano et al. [68]	1	1	1	1	1	0	0	0	1	1	1	8
Narazaki et al. [115]	1	1	1	1	1	0	0	0	1	1	1	8
Pernigoni et al. [116]	1	1	1	1	1	1	0	0	1	1	1	9
Palmer et al. [71]	1	1	1	1	1	0	1	0	1	1	1	9
Palmer et al. [72]	1	1	1	1	1	0	1	0	1	1	1	9
Palmer et al. [73]	1	1	1	1	1	0	1	0	1	1	1	9
Palmer et al. [74]	1	1	1	1	1	0	1	0	1	1	1	9
Peterson et al. [140]	1	1	1	1	1	1	0	0	1	1	1	9
Pino-Ortega et al. [141]	1	1	1	1	1	1	1	0	1	1	1	10
Portes et al. [88]	1	1	1	1	1	1	1	0	1	1	1	10
Portes et al. [89]	1	1	1	1	1	1	1	0	1	1	1	10
Ransdell et al. [142]	1	1	0	1	1	1	1	1	1	1	1	10
Reina et al. [44]	1	1	1	1	1	0	1	1	1	1	1	10
Reina et al. [79]	1	1	0	1	0	1	1	0	1	1	0	7
Reina et al. [97]	1	1	1	1	1	1	1	0	1	1	1	10
Salazar et al. [35]	1	1	1	1	1	0	0	0	1	1	1	8
Salazar et al. [36]	1	1	1	1	1	1	0	0	1	1	1	9
Sampaio et al. [53]	1	1	1	1	1	0	1	0	1	1	1	9
Scanlan et al. [54]	1	1	1	1	1	1	0	0	1	1	1	9
Scanlan et al. [55]	1	1	1	1	1	1	1	0	1	1	1	10
Scanlan et al. [56]	1	1	1	1	1	1	0	0	1	1	1	9
Scanlan et al. [70]	1	1	1	1	1	0	1	0	1	1	1	9
Scanlan et al. [60]	1	1	1	1	1	1	0	0	1	1	1	9
Scanlan et al. [133]	1	1	1	1	1	0	1	0	1	1	1	9
Sosa et al. [85]	1	1	1	1	1	1	0	0	1	1	1	9
Staunton et al. [75]	1	1	1	1	1	1	0	0	1	1	1	9
Staunton et al. [76]	1	1	1	1	1	1	0	0	1	1	1	9
Staunton et al. [77]	1	1	1	1	1	1	0	0	1	1	1	9
Svilar et al. [61]	1	1	1	1	1	0	1	0	1	1	1	9
Svilar et al. [62]	1	1	1	1	1	0	1	0	1	1	1	9
Svilar et al. [37]	1	1	1	1	1	0	1	0	1	1	1	9
Tessitore et al. [143]	1	1	1	1	1	1	0	0	1	1	1	9
Torres-Ronda et al. [117]	1	1	1	1	1	0	1	0	1	1	1	9
Vazquez-Guerrero et al. [80]	1	1	1	1	1	0	1	0	1	1	1	9
Vazquez-Guerrero et al. [94]	1	1	1	1	1	0	1	0	1	1	1	9
Vazquez-Guerrero et al. [81]	1	1	1	1	0	1	1	0	1	1	1	9
Vazquez-Guerrero et al. [82]	1	1	1	1	1	1	1	0	1	1	1	10
Vazquez-Guerrero et al. [95]	1	1	1	1	1	0	1	0	1	1	1	9
Vazquez-Guerrero et al. [83]	1	1	1	1	1	1	1	0	1	1	1	10
Vazquez-Guerrero et al. [84]	1	1	1	1	1	0	1	0	1	1	1	9
Vazquez-Guerrero et al. [63]	1	1	1	1	1	0	0	0	1	1	1	8
Vazquez-Guerrero et al. [96]	1	1	1	1	1	0	0	0	1	1	1	8
Williams et al. [38]	1	1	1	1	1	1	0	0	1	1	1	9
Williams et al. [15]	1	1	1	1	1	1	0	0	1	1	1	9

### Player Characteristics

The characteristics of players monitored in the included studies are presented in Table 4. Sample sizes across studies ranged from six to 104 players. The mean age of players monitored across studies ranged from 14 to 55 years. Considering player sex, 61 studies monitored only male players, 18 studies monitored only female players, and seven studies monitored male and female players in combination. Regarding playing level, some studies included players competing at different playing levels, resulting in seven studies monitoring club players, eight studies monitoring collegiate/university players, 19 studies monitoring representative players, 18 studies monitoring semi-professional players, and 39 studies monitoring professional players.

**Table 4:** Characteristics of the basketball players recruited and the equipment used in the included studies

Study	Year	Participant sex, sample size	Age (years)	Playing level	Monitoring approach	Hardware	Software
Abdelkrim et al. [105]	2010	Male, 38	18.2 ± 0.5	Representative	Time-motion analysis	Sony, DSR-PD170P camera	PC foot v4.0
Abdelkrim et al. [31]	2010	Male, 18	18.2 ± 0.5	Representative	Time-motion analysis	Sony, DSR-PD170P camera	PC Team Sports v4.0
Abdelkrim et al. [5]	2007	Male, 38	18.2 ± 0.5	Representative	Time-motion analysis	Sony, DSR-PD170P camera	PC foot v4.0
Abdelkrim et al. [106]	2020	Female, 30	18.2 ± 0.5	Representative	Time-motion analysis	Sony, HVR-HD1000E camera	PC foot v4.0
Alonso Perez-Chao et al. [39]	2022	Males, 13	16.6 ± 1.0	Representative	Local positioning system	ClearSkyS7	Not specified
Alonso Perez-Chao et al. [40]	2021	Male, 13	16.6 ± 1.0	Representative	Local positioning system	ClearSkyS7	Not specified
Aoki et al. [14]	2017	Male, 9	27.8 ± 6.4	Professional	Microsensors	Zephyr™ Bioharness	Not specified
Arede et al. [86]	2021	Male, 30	13.5 ± 1.2	Representative	Local positioning system	WIMU PRO	Not specified
Arede et al. [90]	2021	Male, 20	19.5 ± 4.4	Representative	Local positioning system	WIMU PRO	Not specified
Ball et al. [57]	2022	Female, 14	24.4 ± 5.8	Professional	Microsensors	OptimEye s5	OpenField
Bishop et al. [51]	2006	Male, 6	Not specified	Professional	Time-motion analysis	Sony DRV900E camera	Nordulus Observer
Bredt et al. [64]	2022	Male, 13 Male, 13 Male, 19	14.7 ± 0.3 14.7 ± 0.3 13.7 ± 0.3	Club	Microsensors	SPI ProX HPU HPU	Not specified
Bredt et al. [65]	2020	Male, 12	17.0 ± 0.2	Club	Microsensors	Delsys Trigno Wireless EMG System	MATLAB R2010a
Castillo et al. [78]	2021	Male, 14	20.0 ± 2.3	Professional	Local positioning system	WIMU PRO	S PRO WIMU
Castillo et al. [87]	2021	Male, 30	15.7 ± 1.7	Club	Local positioning system	WIMU PRO	S PRO WIMU
Conte et al. [107]	2015	Female, 12	27 ± 4	Professional	Time-motion analysis	Sony HD AVCHD HDR-CX115 camera	Dartfish v6.0
Delextrat et al. [108]	2013	Male, 9	22.8 ± 2.2	College/university	Time-motion analysis	JVC- × 400 camera	Not specified
Delextrat et al. [109]	2012	Female, 9	24.3 ± 4.1	Professional	Time-motion analysis	JVC- × 400 camera	Not specified
Ferioli et al. [110]	2020	Male, 44	26.5 ± 4.4	Professional	Time-motion analysis	GoPro Hero 4 (silver edition) camera	SICS VideoMatch Basket v5.0.5
Ferioli et al. [111]	2020	Male, 10	18.3 ± 1.0	Club	Time-motion analysis	GoPro Hero 4 (silver edition) camera	SICS VideoMatch Basket v5.05
Ferioli et al. [112]	2020	Male, 33 Male, 37 Male, 36 Male, 30	27 ± 5 25 ± 4 26 ± 6 22 ± 5	Professional Division I Division II Division III Division IV	Time-motion analysis	GoPro Hero 4 (silver edition) camera	SICS VideoMatch Basket v5.05
Ferioli et al. [138]	2022	Male, 52 Female, 52	Not specified	Professional	Time-motion analysis	Not specified	SICS VideoMatch Basket v5.05
Fox et al. [58]	2020	Male, 8	23.1 ± 3.8	Semi-professional	Microsensors	OptimEye s5	OpenField
Fox et al. [33]	2020	Male, 8	24.4 ± 3.2	Semi-professional	Microsensors	OptimEye s5	OpenField
Fox et al. [34]	2019	Male, 5	24.4 ± 3.2	Semi-professional	Microsensors	OptimEye s5	OpenField
Fox et al. [32]	2021	Male, 8	23.1 ± 3.8	Semi-professional	Microsensors	OptimEye s5	OpenField
Fox et al. [59]	2021	Male, 8	23 ± 4	Semi-professional	Microsensors	OptimEye s5	OpenField
Garcia et al. [91]	2020	Male, 13	19.8 ± 1.7	Professional	Local positioning system	WIMU PRO	S PRO WIMU
Garcia et al. [92]	2021	Male, 64	15.0 ± 2.9	Professional and representative	Local positioning system	WIMU PRO	S PRO WIMU
Garcia et al. [41]	2022	Male, 14	20.6 ± 2.7	Professional	Local positioning system	WIMU PRO	S PRO WIMU
Garcia et al. [42]	2022	Male, 12	19.6 ± 1.7	Professional	Local positioning system	WIMU PRO	S PRO WIMU
Garcia et al. [43]	2021	Male, 10	20 ± 1.5	Professional	Local positioning system	WIMU PRO	S PRO WIMU
Garcia et al. [93]	2021	Male, 13	19.8 ± 1.7	Professional	Local positioning system	WIMU PRO	S PRO WIMU
Heishman et al. [139]	2020	Male, 14	19.7 ± 1.0	College/university	Microsensors	OptimeEye T6	OpenField
Hulka et al. [52]	2013	Male, 32	16.9 ± 0.7	Representative	Time-motion analysis	Canon HF10	Video Manual Motion Tracker v1.0
Ibanez et al. [45]	2022	Female, 22	22.5 ± 2.7	Professional	Local positioning system	WIMU PRO	S PRO WIMU
Klusemann et al. [113]	2013	Male, 8	17.8 ± 0.2	Representative	Time-motion analysis	Not specified	SportsCode Elite
Koyama et al. [66]	2020	Male, 18	19.5 ± 0.8	College/university	Microsensors	OptimEye S5	Sports Sensing and Dartfish
Koyama et al. [67]	2022	Male, 21	20.0 ± 1.0	College/university	Microsensors	OptimEye S5	Not specified
Leicht et al.[69]	2019	Male, 6	23.5 ± 3.6	Semi-professional	Microsensors	OptimEye s5	OpenField
Matthew et al. [114]	2009	Female. 9	25.8 ± 2.5	College/university	Time-motion analysis	JVC- × 400 camera	Not specified
McInnes et al. [104]	1995	Male, 8	Not listed	Professional	Time-motion analysis	National M-7 camera	Not specified
Nagano et al. [68]	2021	Female, 18	16.1 ± 0.6	Representative	Microsensors	SS-WS1201	Dartfish
Narazaki et al. [115]	2009	Male, 6 Female, 6	20.8 ± 1.0 20.0 ± 1.3	College/university	Time-motion analysis	Canon ZR20 camera	Not specified
Palmer et al. [71]	2021	Female, 12 Male, 13 Female, 12	25.2 ± 5.9 26.8 ± 5.2 28.1 ± 5.0	Professional Semi-professional Semi-professional	Microsensors	GT9X Actigraph	Actilife v6.13.4
Palmer et al. [72]	2022	Female, 10 Male, 12	28.5 ± 5.4 26.8 ± 5.2	Semi-professional Semi-professional	Microsensors	GT9X Actigraph	Actilife v6.13.4
Palmer et al. [73]	2022	Female, 12 Female, 12 Male 13	25.2 ± 5.9 28.1 ± 5.0 26.8 ± 5.2	Professional Semi-professional Semi-professional	Microsensors	GT9X Actigraph	Actilife v6.13.4
Palmer et al. [74]	2022	Female, 12	25.2 ± 5.9	Professional	Microsensors	GT9X Actigraph	Actilife v6.13.4
Pernigoni et al. [116]	2021	Male, 11	20.5 ± 1.1	Professional	Time-motion analysis	Redmi 5 Plus	PotPlayer
Peterson et al. [140]	2017	Female, 5	20 ± 1.0	College/university	Microsensors	OptimEye S5	Openfield
Pino-Ortega et al. [141]	2019	Male, 94	17.6 ± 0.8	Representative	Local positioning system	WIMU PRO	S PRO WIMU
Portes et al. [88]	2020	Male, 25 Female, 48	17 ± 1 17 ± 1	Representative Representative	Local positioning system	WIMU PRO	S PRO WIMU
Portes et al. [89]	2022	Female, 48	16.8 ± 0.7	Representative	Local positioning system	WIMU PRO	S PRO WIMU
Ransdell et al. [142]	2020	Female, 6	19.7 ± 1.5	College/university	Microsensors	OptimEye S5	OpenField
Reina et al. [44]	2020	Female, 48	Not specified	Club	Local positioning system	WIMU PRO	S PRO WIMU
Reina et al. [79]	2020	Female, 48	Not specified	Club	Local positioning system	WIMU PRO	S PRO WIMU
Reina et al. [97]	2019	Female, 48	Not specified	Club	Local positioning system	WIMU PRO	S PRO WIMU
Salazar et al. [35]	2020	Male, 17	27.5 ± 6	Professional	Microsensors	Catapult T6	OpenField
Salazar et al. [36]	2021	Male, 11	16.7 ± 0.7	Representative	Microsensors	Catapult T6	OpenField
Sampaio et al. [53]	2016	Male, 20	16.1 ± 2.1	Semi-professional	Time-motion analysis	Not specified	Simi Scout v2.0.0.174
Scanlan et al. [54]	2011	Male, 10 Male, 12	28.3 ± 4.9 26.1 ± 5.3	Professional Semi-Professional	Time-motion analysis	JVC Everio GZ-HD10 camera Basler A602FC camera	Labview
Scanlan et al. [55]	2015	Male, 12 Female, 12	26.1 ± 5.3 22.0 ± 3.7	Semi-professional	Time-motion analysis	Basler A602FC camera	Labview
Scanlan et al. [56]	2012	Female, 12	22.0 ± 3.7	Semi-professional	Time-motion analysis	Basler A602FC camera	Labview
Scanlan et al. [70]	2019	Male, 13	20.4 ± 4.6	Semi-professional	Microsensors	OptimEye S5	Catapult Sprint
Scanlan et al. [60]	2019	Male, 5	24 ± 3	Semi-professional	Microsensors	OptimEye S5	OpenField
Scanlan et al. [133]	2015	Male, 10 Male, 12	28.3 ± 4.9 26.1 ± 5.3	Professional Semi-professional	Time-motion analysis	JVC Everio GZ-HD10 camera Basler A602FC camera	Labview
Sosa et al. [85]	2021	Male, 10	16.9 ± 1.1	Representative	Local positioning system	ClearSkyS7	OpenField
Staunton et al. [75]	2018	Female, 9	27.0 ± 5.0	Professional	Microsensors	ActiGraph	ActiLife v12
Staunton et al. [76]	2018	Female, 10	27.0 ± 5.0	Professional	Microsensors	ActiGraph	ActiLife v12
Staunton et al. [77]	2020	Female, 9	26.0 ± 3.0	Professional	Microsensors	ActiGraph	ActiLife v12
Svilar et al. [61]	2018	Male, 13	25.7 ± 3.3	Professional	Microsensors	Catapult S5	OpenField v1.14.0
Svilar et al. [62]	2019	Male, 16	26.2 ± 4.0	Professional	Microsensors	Catapult T6	OpenField v1.17
Svilar et al. [37]	2018	Male, 13	26.3 ± 2.2	Professional	Microsensors	Catapult S5	OpenField v1.14.0
Tessitore et al. [143]	2006	Male, 10	55 ± 5	Representative	Time-motion analysis	JVC DL 107	Video recorder JVC BR 8600
Torres-Ronda et al. [117]	2016	Male, 14	25.5 ± 4.7	Professional	Time-motion analysis	Not specified	Lince sport analysis software
Vazquez-Guerrero et al. [80]	2020	Male, 94	17.4 ± 0.7	Representative	Local positioning system	WIMU PRO	S PRO WIMU
Vazquez-Guerrero et al. [94]	2020	Male, 12	29.6 ± 4.5	Professional	Local positioning system	WIMU PRO	S PRO WIMU
Vazquez-Guerrero et al. [81]	2019	Male, 94	17.4 ± 0.7	Professional	Local positioning system	WIMU PRO	S PRO WIMU
Vazquez-Guerrero et al. [82]	2019	Male, 94	17.4 ± 0.74	Professional	Local positioning system	WIMU PRO	S PRO WIMU
Vazquez-Guerrero et al. [95]	2020	Male, 21	27.9 ± 3.9	Professional	Local positioning system	WIMU PRO	S PRO WIMU
Vazquez-Guerrero et al. [83]	2019	Male, 94	17.4 ± 0.7	Professional	Local positioning system	WIMU PRO	S PRO WIMU
Vazquez-Guerrero et al. [84]	2018	Male, 12	29.6 ± 4.5	Professional	Local positioning system	WIMU PRO	S PRO WIMU
Vazquez-Guerrero et al. [63]	2018	Male, 12	25.5 ± 5.2	Professional	Microsensors	ADXL326	Viper v2.6.0.0
Vazquez-Guerrero et al. [96]	2021	Male, 12	29.6 ± 4.5	Professional	Local positioning system	WIMU PRO	S PRO WIMU
Williams et al. [38]	2021	Male, 8	24.3 ± 3.9	Semi-professional	Microsensors	OptimEyeS5	OpenField
Williams et al. [15]	2022	Male, 7	23 ± 4	Semi-professional	Microsensors	OptimEyeS5	OpenField

### Video-Based Time-Motion Analysis: Non-numeric Thresholds to Identify Intensity Zones

Studies utilizing video-based TMA with non-numeric thresholds to identify intensity zones (*n* = 18) are presented in Table 5. Across studies, 10 different cameras were used for filming, with three studies not specifying the camera used for filming. Further, 12 studies stated the software utilized to analyze recorded video, with seven different software programs utilized across studies. Moreover, six studies did not specify any software to analyze video or replayed footage on a video recorder.

**Table 5:** Intensity zones from included studies utilizing non-numeric thresholds for video-based time-motion analysis

Study	Number of intensity zones	Locomotion-based movement intensity zones	Basketball-specific movement intensity zones
Abdelkrim et al. [105]	Total: 11 zones Locomotion: 5 zones Basketball-specific: 6 zones	Stand, walk, jog, run, sprint	Low shuffle, moderate shuffle, high shuffle, jump, pick, positioning
Abdelkrim et al. [5]	Total: 9 zones Locomotion: 5 zones Basketball-specific: 4 zones	Stand, walk, jog, run, sprint	Low specific movement, moderate specific movement, high specific movement, jump
Abdelkrim et al. [106]	Total: 11 zones Locomotion: 5 zones Basketball-specific: 6 zones	Stand, walk, jog, run, sprint	Low shuffling, moderate shuffling, high shuffling, jump, screen/pick, positioning
Bishop et al. [51]	Total: 3 zones	Low-intensity, medium-intensity, high-intensity (all movements grouped together)	Not included
Conte et al. [107]	Total: 10 zones Locomotion: 4 zones Basketball-specific: 6 zones	Stand/walk, jog, run, sprint	Low-intensity specific movement, moderate-intensity specific movement, high-intensity specific movement, jump, pick, positioning
Delextrat et al. [108]	Total: 6 zones Locomotion: 4 zones Basketball-specific: 2 zones	Stand/walk, jog, run, sprint	Shuffle, jump
Delextrat et al. [109]	Total: 8 zones Locomotion: 4 zones Basketball-specific: 4 zones	Stand/walk, jog, run, sprint	Low-intensity shuffle, medium-intensity shuffle, high-intensity shuffle, jump
Ferioli et al. [110]	Total: 8 zones Locomotion: 4 zones Basketball-specific: 4 zones	Stand/walk, jog (forward or backward), run (forward or backward), sprint (forward or backward)	Low specific movement, moderate specific movement, high specific movement, jump
Ferioli et al. [111]	Total: 8 zones Locomotion: 4 zones Basketball-specific: 4 zones	Stand/walk, jog (forward or backward), run (forward or backward), sprint (forward or backward)	Low specific movement, moderate specific movement, high specific movement, jump
Ferioli et al. [112]	Total: 8 zones Locomotion: 4 zones Basketball-specific: 4 zones	Stand/walk, jog (forward or backward), run (forward or backward), sprint (forward or backward)	Low specific movement, moderate specific movement, high specific movement, jump
Ferioli et al. [138]	Total: 8 zones Locomotion: 4 zones Basketball-specific: 4 zones	Stand/walk, jog (forward or backward), run (forward or backward), sprint (forward or backward)	Low specific movement, moderate specific movement, high specific movement, jump
Klusemann et al. [113]	Total: 8 zones Locomotion: 4 zones Basketball-specific: 4 zones	Stand/walk, jog, run, sprint	Low-intensity shuffle, medium-intensity shuffle, high-intensity shuffle, jump
Matthew et al. [114]	Total: 8 zones Locomotion: 4 zones Basketball-specific: 4 zones	Stand/walk, jog, run, sprint	Low-intensity shuffle, medium-intensity shuffle, high-intensity shuffle, jump
McInnes et al. [104]	Total: 9 zones Locomotion: 5 zones Basketball-specific: 4 zones	Stand/walk, jog, run, run backwards, stride/sprint	Low shuffle, medium shuffle, high shuffle, jump
Narazaki et al. [115]	Total: 4 zones Locomotion: 3 zones Basketball-specific: 1 zone	Stand, walk, run	Jump
Pernigoni et al. [116]	Total: 3 zones Locomotion: 1 zone Basketball-specific: 2 zones	Sprint	High-intensity specific movement, jump
Tessitore et al. [143]	Total: 5 zones Locomotion: 3 zones Basketball-specific: 2 zones	Inactivity, walk, run	Jump, positioning
Torres-Ronda et al. [117]	Total: 11 zones Locomotion: 4 zones Basketball-specific: 7 zones	Stand, walk, jog/run, sprint	Low-intensity specific movement, moderate-intensity specific movement, high-intensity specific movement, jump, screen/pick, positioning, static

Movement zones were delineated into locomotion-based movements and basketball-specific movements. Locomotion-based movements were defined as standing and any ambulation strategies ranging in intensity from walking to sprinting with a mean of 3.9 ± 1.0 (range 3–5) zones reported across studies. Basketball-specific movements were defined as non-locomotion-based movements that are commonly performed during basketball gameplay, including jumping, shuffling, dribbling, and screening/picking, with a mean of 4.0 ± 1.6 (range 1–7) zones reported across studies. A mean of 7.7 ± 2.5 (range 3–11) total movement zones (locomotion-based and basketball-specific movements combined) were reported across studies. One study [51] combined all locomotion-based and basketball-specific movements together and thus was not included in the mean zone calculations for each of these categories given they were not included.

### Video-Based Time-Motion Analysis: Numeric Thresholds to Identify Intensity Zones

Studies utilizing video-based TMA with numeric thresholds to identify intensity zones (*n* = 6) are presented in Table 6. Across all six studies, four different cameras were used for filming, with one study not specifying the camera used for filming. All studies stated the software utilized to analyze recorded video, with four different software programs being utilized across studies. Across studies, the mean total intensity zones used was 7.3 ± 2.9 (range 3–11), the mean locomotion-based movement intensity zones used was 4.3 ± 0.5 (range 4–6), and the mean basketball-specific movement intensity zones used was 3.3 ± 2.4 (range 0–5). One study [52] combined all locomotion-based and basketball-specific movements together and thus was not included in the mean zone calculations for each of these categories given they were not included.

**Table 6:** Intensity zones from included studies utilizing numeric thresholds for video-based time-motion analysis

Study	Intensity zones (thresholds in m·s^−1^)										
	Locomotion-based movement intensity zones						Basketball-specific movement intensity zones				
Abdelkrim et al. [31]^a^	Stand (0)	Walk (≤ 1.7)	Jog (1.7–3.3)	Run (3.4–5.0)	Stride (5.0–6.7)	Sprint (> 6.7)	Low shuffle (≤ 1.7)	Moderate shuffle (1.7–2.5)	High shuffle (> 2.5)	Sideways run (> 3.3)	Jump
Hulka et al. [52]	Low-intensity (0–3.0)	Medium-intensity (3.10–5.0)		High-intensity (> 5.1)							
Sampaio et al. [53]	Stand (0.0–0.1)	Walk (0.2–2.0)	Jog (2.1–3.7)	Run (3.8–6.0)	Sprint (> 6.1)						
Scanlan et al. [54]	Stand/walk (0–1.0)	Jog (1.1–3.0)	Run (3.1–7.0)	Sprint (> 7.0)			Low shuffle (≤ 2.0)	High shuffle (> 2.0)	Jump	Dribble	Upper-body
Scanlan et al. [55]	Stand/walk (< 1.0)	Jog (1.1–3.0)	Run (3.1–7.0)	Sprint (> 7.0)			Low shuffle (≤ 2.0)	High shuffle (> 2.0)	Dribble		
Scanlan et al. [56]	Stand/walk (0–1.0)	Jog (1.1–3.0)	Run (3.1–7.0)	Sprint (> 7.0)			Low shuffle (≤ 2.0)	High shuffle (> 2.0)	Jump	Dribble	Upper-body

^a^Thresholds that were reported in km × h^−1^ and converted to m·s^−1^ via multiplying values by 5/18 for consistency in units reported across studies using this technology

Across studies (*n* = 6), standing and walking were considered as separate intensity zones in two studies, where thresholds used to identify standing were 0 m·s^−1^ [31] and 0–0.1 m·s^−1^ [53], and thresholds used to identify walking were < 1.7 m·s^−1^ [31] and 0.2–2.0 m·s^−1^ [53]. In contrast, three studies [54–56] included walking and standing together in the same intensity zone, with thresholds of 0 m·s^−1^ to 1.0 m·s^−1^ used consistently. Further, one study [52] grouped all low-intensity movements (i.e., inactivity, walking and jogging) as < 3.0 m·s^−1^.

Across the six studies using video-based TMA with numeric thresholds, five of them [31, 53–56] assigned intensity zone thresholds to jogging, running, and sprinting activity. The mean intensity zone thresholds assigned to jogging were 1.4 ± 0.5 m·s^−1^ (range 1.1–2.1 m·s^−1^) to 3.2 ± 0.3 m·s^−1^ (range 3.0–3.7 m·s^−1^) across studies. The mean intensity zone thresholds assigned to running were 3.3 ± 0.3 m·s^−1^ (range 3.1–3.8 m·s^−1^) to 6.4 ± 0.9 m·s^−1^ (range 5.0–7.0 m·s^−1^) across studies. Additionally, one study [31] included striding as a locomotion-based movement intensity zone positioned between running and sprinting using thresholds of 5.0–6.7 m·s^−1^. Intensity zone thresholds for sprinting involved only an initial threshold with a mean of 6.8 ± 0.4 m·s^−1^ (range > 6.1 to > 7.0 m·s^−1^) across studies. A further study [52] combined all moderate- (3.1–5.0 m·s^−1^) and high-intensity (> 5.10 m·s^−1^) movements into separate categories.

Basketball-specific movement intensity zones were included in four studies, with three of them including intensity thresholds for low- and high-intensity shuffling activity [54–56] and one study [31] including intensity thresholds for low-, medium-, and high-intensity shuffling. Intensity zone thresholds for low-intensity shuffling involved only an upper threshold, with a mean of ≤ 1.9 ± 0.1 m·s^−1^ (range 1.7–2.0 m·s^−1^) across studies. The medium-intensity shuffling intensity zone utilized in a study [31] had thresholds of 1.7–2.5 m·s^−1^, with this study also including sideways running with a single threshold of > 3.3 m·s^−1^ as an additional basketball-specific movement. Intensity zone thresholds for high-intensity shuffling included only an initial threshold with a mean of > 2.1 ± 0.2 m·s^−1^ (range > 2.0 to > 2.5 m·s^−1^) across studies. In addition, four studies contained movements that did not have numeric intensity thresholds including jumping [31, 54, 56], dribbling [54–56], and upper-body movements [54, 56].

### Microsensors

Studies utilizing microsensors (*n* = 33) to determine intensity zones are detailed in Table 7. Across studies, eight different microsensors were used to collect data and seven different software programs were used to analyze data, with three studies not specifying the software used to analyze data. The external load variables and the intensity zones used for each variable differed across studies. The mean total number of intensity zones (i.e., number of intensity zones defined) was 2.4 ± 2.1 (range 1–11) across all studies. In turn, 1.9 ± 1.1 (range 1–4) acceleration intensity zones were utilized across 20 studies, 1.5 ± 0.9 (range 1–3) deceleration intensity zones were utilized across 14 studies, 5.7 ± 0.6 (range 5–6) PlayerLoad intensity zones were utilized across three studies, 1.4 ± 0.9 (range 1–3) jump intensity zones were utilized across 13 studies, 1.8 ± 1.0 (range 1–3) change-of-direction (COD) intensity zones were utilized across eight studies, and 5.9 ± 1.1 (range 5–7) average net force intensity zones were utilized across seven studies.

**Table 7:** Intensity zones from included studies utilizing microsensors

Study	Variable (unit)	Intensity zone thresholds						
Ball et al. [57]	Acceleration (m·s^−2^)	≥ 2.5						
Bredt et al. [64]	Acceleration (G)	0–0.5	0.5–1.0	1.0–1.5	1.5–2.0			
Bredt et al. [65]	Acceleration (G)	0–0.5	> 0.5–1.0	> 1.0–1.5	> 1.5–2.0			
Fox et al. [58]	Acceleration (m·s^−2^)	1.5–2.5	2.5–3.5	> 3.5				
	Deceleration (m·s^−2^)	1.5–2.5	2.5–3.5	> 3.5				
	COD (m·s^−2^)	1.5–2.5	2.5–3.5	> 3.5				
	Jump (cm)	< 20	20–40	> 40				
Fox et al. [33]	Acceleration (m·s^−2^)	> 3.5						
	Deceleration (m·s^−2^)	> 3.5						
	Jump (cm)	> 40						
Fox et al. [34]	Acceleration (m·s^−2^)	> 3.5						
	Deceleration (m·s^−2^)	> 3.5						
	Jump (cm)	> 40						
Fox et al. [32]	Acceleration (m·s^−2^)	> 3.5						
	Deceleration (m·s^−2^)	> 3.5						
	Jump (cm)	> 40						
Fox et al. [59]	Acceleration (m·s^−2^)	1.5–2.5	2.5–3.5	> 3.5				
	Deceleration (m·s^−2^)	1.5–2.5	2.5–3.5	> 3.5				
	COD (m·s^−2^)	1.5–2.5	2.5–3.5	> 3.5				
	Jump (cm)	< 20	20–40	> 40				
Heishman et al. [139]	IMA (m·s^−2^)	1.5–2.5	2.5–3.5	> 3.5				
Koyama et al. [66]	Acceleration (G)	> 4	> 6	> 8				
Koyama et al. [67]	Acceleration (G)	> 4	> 6	> 8				
Leicht et al. [69]	PlayerLoad (AU)	0–1	1–2	2–3	3–4	4–6		
Nagano et al. [68]	Accelerations (G)	> 6	> 8					
Palmer et al. [71]	AvF_Net_ (%VO_2_R)	≤ 10	> 10–40	> 40–90	> 90–100	> 100		
Palmer et al. [72]	AvF_Net_ (%VO_2_R)	≤ 10	> 10–40	> 40–90	> 90–100	> 100		
Palmer et al. [73]	AvF_Net_ (%VO_2_R)	≤ 10	> 10–40	> 40–90	> 90–100	> 100		
Palmer et al. [74]	AvF_Net_ (%VO_2_R)	≤ 10	> 10–40	> 40–90	> 90–100	> 100		
Peterson et al. [140]	Anterior–posterior IMA (m·s^−2^)	≥ 3.5						
	Medial–lateral IMA (m·s^−2^)	≥ 3.5						
	Vertical IMA (cm)	≥ 25.4						
Ransdell et al. [142]	IMA (m·s^−2^)	≥ 3.5						
Salazar et al. [35]	Acceleration (m·s^−2^)	> 3.5						
	Deceleration (m·s^−2^)	> 3.5						
	Jump (cm)^a^	> 40						
Salazar et al. [36]	Acceleration (m·s^−2^)	> 3.0						
	Deceleration (m·s^−2^)	< − 3.0						
	Jump (cm)	> 40						
Scanlan et al. [70]	PlayerLoad (AU)	0–1	1–2	2–3	3–4	4–6	6–10	
	Relative PlayerLoad (%)	0–10	10–20	20–30	30–40	40–60	60–100	
Scanlan et al. [60]	PlayerLoad (AU)	0–1	1–2	2–3	3–4	4–6	6–10	
	Acceleration (m·s^−2^)	1.5–2.5	2.5–3.5	> 3.5				
	Deceleration (m·s^−2^)	1.5–2.5	2.5–3.5	> 3.5				
	COD (m·s^−2^)	1.5–2.5	2.5–3.5	> 3.5				
	Jump (cm)	< 20	20–40	> 40				
Staunton et al. [75]	AvF_Net_ (% VO_2_R)	< 20	20 to < 30	30 to < 40	40 to < 60	60 to < 90	90 to < 100	≥ 100
Staunton et al. [76]	AvF_Net_ (% VO_2_R)	< 20	20 to < 30	30 to < 40	40 to < 60	60 to < 90	90 to < 100	≥ 100
Staunton et al. [77]	AvF_Net_ (% VO_2_R)	< 20	20 to < 30	30 to < 40	40 to < 60	60 to < 90	90 to < 100	≥ 100
Svilar et al. [61]	Acceleration (m·s^−2^)	> 3.5						
	Deceleration (m·s^−2^)	< − 3.5						
	COD (m·s^−2^)	> 3.5						
	Jump (cm)^a^	> 40						
Svilar et al. [62]	Acceleration (m·s^−2^)	> 3.5						
	Deceleration (m·s^−2^)	< − 3.5						
	COD (m·s^−2^)	< − 3.5						
	Jump (cm)	> 40						
Svilar et al. [37]	Acceleration (m·s^−2^)	> 3.5						
	Deceleration (m·s^−2^)	> 3.5						
	COD (m·s^−2^)	> 3.5						
	Jump (cm)^a^	> 40						
Vazquez-Guerrero et al. [63]	Acceleration (m·s^−2^)	< 3	> 3					
	Deceleration (m·s^−2^)	< 3	> 3					
Williams et al. [38]	Acceleration (m·s^−2^)	> 3.5						
	Deceleration (m·s^−2^)	> 3.5						
	COD (m·s^−2^)	> 3.5						
	Jump (cm)	> 40						
Williams et al. [15]	Acceleration (m·s^−2^)	> 3.5						
	Deceleration (m·s^−2^)	> 3.5						
	COD (m·s^−2^)	> 3.5						
	Jump (cm)	> 40						

^a^Thresholds were reported in m and converted to cm via multiplying values by 100 for consistency in units across studies using this technology. One study conducted by Aoki et al. [14] is not shown for ease of presentation but used 11 intensity zones for mechanical load (AU) including thresholds of 0–0.50, 0.50–0.75, 0.75–1.00, 1.00–1.25, 1.25–1.50, 1.50–1.75, 1.75–2.0, 2.0–2.25, 2.25–2.50, 2.50–2.75, and 2.75–3.00

*G* g-force, *COD* changes in direction, *IMA* inertial movement analysis, *AU* arbitrary units, *VO*_*2*_*R* oxygen uptake reserve

In studies that measured accelerations (*n* = 20), 15 studies [15, 32–38, 57–63] used m·s^−2^ while five studies [64–68] used g-forces as the measurement unit (Table 7). In the 15 studies using m·s^−2^, 11 studies [15, 32–38, 57, 61, 62] adopted only an initial threshold to identify high-intensity accelerations, with a mean of > 3.3 ± 0.3 m·s^−2^ (range > 2.5 to > 3.5 m·s^−2^) across studies. Mean ± SD thresholds were not able to be calculated across studies using g-forces due to the wide variation in intensity zones adopted. All studies measuring decelerations (*n* = 14) utilized m·s^−2^ as the measurement unit, of which 10 studies [15, 32–38, 61, 62] only cited an initial threshold to identify high-intensity decelerations with a mean of <  − 3.5 ± 0.2 m·s^−2^ (range <  − 3.0 to <  − 3.5 m·s^−2^) across studies. In turn, three studies [58–60] included low-, medium-, and high-intensity zones separately for accelerations and decelerations, with identical thresholds consisting of 1.5–2.5 m·s^−2^, 2.5–3.5 m·s^−2^, and > 3.5 m·s^−2^, respectively, while one study [63] included low- and high-intensity zones separately for accelerations and decelerations with thresholds of < 3.0 m·s^−2^ and > 3.0 m·s^−2^, respectively.

All studies (*n* = 3) [60, 69, 70] including intensity zones for PlayerLoad used arbitrary units (AU) as the measurement unit. Five intensity zones were consistently employed across all studies [60, 69, 70] with thresholds of 0–1 AU, 1–2 AU, 2–3 AU, 3–4 AU, and 4–6 AU, with two studies [60, 70] also including a sixth intensity zone with thresholds of 6–10 AU. Further, a study [70] calculated PlayerLoad intensity zones relative (%) to the individualized peak instantaneous PlayerLoad with thresholds of 0–10%, 10–20%, 20–30%, 30–40%, 40–60%, and 60–100% of the peak PlayerLoad achieved during any training session or game across the study being assigned to each player.

All 13 studies [15, 32–38, 58–62] that measured jumps utilized cm as the measurement unit. In this regard, ten studies [15, 32–38, 61, 62] provided only an initial threshold (> 40 cm) to identify high-intensity jumps, while three studies [58–60] incorporated three intensity zones with thresholds of < 20 cm, 20–40 cm, and > 40 cm used to represent low-, medium-, and high-intensity jumps, respectively.

All eight studies [15, 37, 38, 58–62] that measured COD utilized m·s^−2^ as the measurement unit. In this regard, five studies [15, 37, 38, 61, 62] provided only an initial threshold (> 3.5 m·s^−2^) to identify high-intensity COD while three studies [58–60] incorporated three intensity zones with thresholds of 1.5–2.5 m·s^−2^, 2.5–3.5 m·s^−2^, and > 3.5 m·s^−2^ to represent low-, medium-, and high-intensity COD, respectively.

All seven studies [71–77] that measured average net force used relative (%) intensity zones on the basis of individualized oxygen uptake reserve (VO_2_R) calculated from metabolic measurements taken at rest and during a modified Yo–Yo Intermittent Recovery Test. In turn, four studies [71–74] used five intensity zones consisting of ≤ 10% (inactive), > 10–40% (light), > 40–90% (moderate–vigorous), > 90–100% (maximal), and > 100% (supramaximal), while three studies [75–77] used seven intensity zones consisting of < 20% (sedentary), 20% to < 30% (very light), 30–40% (light), 40% to < 60% (moderate), 60% to < 90% (vigorous), 90% to < 100% (maximal), and ≥ 100% (supramaximal).

### Local positioning systems (LPS)

Studies utilizing LPS (*n* = 25) to determine speed-based intensity zones are presented in Table 8. All studies used km × h^−1^ as the measurement unit for speed-based intensity zones, with a mean of 3.1 ± 1.8 (range 1–5) zones across studies. Studies utilizing LPS (*n* = 22) to determine acceleration and deceleration intensity zones are presented in Table 9. All studies utilized m·s^−2^ as the measurement unit for acceleration and deceleration zones, with a mean of 1.4 ± 1.0 (range 1–5) zones used each for accelerations and decelerations across studies. Across all studies, two different LPS were used to collect data and two different software programs were used to analyze data.

**Table 8:** Speed-based intensity zones from included studies utilizing local positioning systems

Study	Intensity zones (thresholds in km × h^−1^)				
Alonso Perez-Chao et al. [39]	Stand/walk (< 7.0)	Jog (7.01–14.0)	Run (14.01–18.0)	High-speed run (> 18.01)	
Alonso Perez-Chao et al. [40]	Stand/walk (< 7.0)	Jog (7.01–14.0)	Run (14.01–18.0)	High-speed run (> 18.01)	
Arede et al. [86]	Zone 1 (< 6.0)	Zone 2 (6.1–12.0)	Zone 3 (> 18.0)		
Castillo et al. [78]	Walk (< 6.0)	Jog (6.1–12.0)	Cruise (12.1–18.0)	High-speed run (18.1–24.0)	Sprint (> 24.1)
Castillo et al. [87]	Cruise (12.1–18.0)	High-speed run (18.1–24.0)	Sprint (> 24.1)		
Garcia et al. [91]	High-speed run (> 18.0)				
Garcia et al. [92]	High-speed run (> 18.0)				
Garcia et al. [41]	High-speed run (> 18.0)				
Garcia et al. [42]	High-speed run (> 18.0)				
Garcia et al. [43]	> 18.0	> 21.0			
Garcia et al. [93]	> 18.0				
Ibanez et al. [45] *First division* *Second division* *Combined*	Very low/standing < 2.14 < 2.35 < 2.31	Walk 2.14–4.93 2.35–5.16 2.31–5.33	Jog 4.94–8.71 5.17–8.99 5.34–9.32	High/run 8.72–12.55 9.00–12.76 9.33–13.12	Very high/sprint 12.56–16.54 12.77–16.71 13.13–17.08
Pino-Ortega [141]	High-intensity run (> 16.0)				
Portes et al. [88]	High-intensity (14.0–21.0)	Sprint (> 21.0)			
Portes et al. [89]	High-intensity (14.0–21.0)	Sprint (21.0–31.0)			
Reina et al. [44]	High-speed (> 14.4)				
Reina et al. [79]	Stand (< 3.6)	Walk (3.6–6.5)	Jog (6.5–10.2)	Run (10.2–14.4)	Sprint (> 14.4)
Sosa et al. [85]	Stand/walk (< 7.0)	Jog (7.0–14.0)	Run (14.0–18.0)	High-speed run (> 18.0)	
Vazquez-Guerrero et al. [80]	Stationary/walk (< 6.0)	Jog (6.0–12.0)	Run (12.1–18.0)	High-intensity run (18.1–24.0)	Sprint (> 24.1)
Vazquez-Guerrero et al. [81]	Stationary/walk (< 6.0)	Jog (6.0–12.0)	Run (12.1–18.0)	High-intensity run (18.1–24.0)	Sprint (> 24.1)
Vazquez-Guerrero et al. [82]	Stationary/walk (< 6.0)	Jog (6.0–12.0)	Run (12.1–18.0)	High-intensity run (18.1–24.0)	Sprint (> 24.1)
Vazquez-Guerrero et al. [95]	High-speed run (> 18.0)				
Vazquez-Guerrero et al. [83]	Stationary/walk (< 6.0)	Jog (6.0–12.0)	Run (12.1–18.0)	High-intensity run (18.1–24.0)	Sprint (> 24.1)
Vazquez-Guerrero et al. [84]	Stationary/walk (< 6.0)	Jog (6.0–12.0)	Run (12.1–18.0)	High-intensity run (18.1–24.0)	Sprint (> 24.1)
Vazquez-Guerrero et al. [96]	High-speed run (> 18.0)

**Table 9:** Acceleration-based intensity zones from included studies utilizing local positioning systems

Study	Intensity zones (thresholds in m·s^−2^)	
	Acceleration	Deceleration
Alonso Perez-Chao [39]	> 2.0	> 2.0
Alonso Perez-Chao [40]	> 2.0	< 2.0
Arede et al. [86]	> 2.0	> − 2.0
Arede et al. [90]	≥ 2.0	> − 2.0
Castillo et al. [78]	Zone 1: < 2.0	Zone 1: < − 2.0
	Zone 2: > 2.0	Zone 2: > − 2.0
Castillo et al. [87]	> 0	< 0
Garcia et al. [91]	≥ 2.0	≤ − 2.0
Garcia et al. [92]	≥ 2.0	≤ − 2.0
Garcia et al. [41]	> 3.0	> 3.0
Garcia et al. [42]	> 3.0	> 3.0
Garcia et al. [43]	Zone 1: > 2.0	Zone 1: > 2.0
	Zone 2: > 3.0	Zone 2: > 3.0
Garcia et al. [93]	≥ 2.0	≤ − 2.0
Ibanez et al. [45]	*First division* Zone 1: < 0.58 Zone 2: 0.58–1.86 Zone 3: 1.87–3.21 Zone 4: 3.22–4.58 Zone 5: 4.59–7.01 *Second division* Zone 1: < 0.44 Zone 2: 0.44–1.44 Zone 3: 1.45–2.64 Zone 4: 2.65–3.65 Zone 5: 3.66–4.61 *Combined* Zone 1: < 0.5 Zone 2: 0.50–1.60 Zone 3: 1.61–2.87 Zone 4: 2.88–4.25 Zone 5: 4.26–6.71	*First division* Zone 1: > − 0.62 Zone 2: − 0.62 to − 2.00 Zone 3: − 2.01 to − 3.42 Zone 4: − 3.43 to − 4.86 Zone 5: − 4.87 to − 6.51 *Second division* Zone 1: > − 0.52 Zone 2: − 0.52 to − 1.74 Zone 3: − 1.75 to − 3.01 Zone 4: − 3.02 to − 4.22 Zone 5: − 4.23 to − 5.65 *Combined* Zone 1: > − 0.37 Zone 2: − 0.37 to − 1.13 Zone 3: − 1.14 to − 2.07 Zone 4: − 2.08 to − 3.23 Zone 5: − 3.24 to − 4.77
Reina et al. [44]	> 2.5	> 2.5
Reina et al. [97]	Zone 1: 1.0–2.5	Zone 1: − 1.0 to − 2.5
	Zone 2: 2.5–4.0	Zone 2: − 2.5 to − 4.0
	Zone 3: > 4.0	Zone 3: > − 4
Vazquez-Guerrero et al. [80]	> 2.0	< − 2.0
Vazquez-Guerrero et al. [94]	> 2.0	< − 2.0
Vazquez-Guerrero et al. [82]	> 2.0	< − 2.0
Vazquez-Guerrero et al. [95]	> 2.0	< − 2.0
Vazquez-Guerrero et al. [83]	> 2.0	< − 2.0
Vazquez-Guerrero et al. [84]	> 2.0	< − 2.0
Vazquez-Guerrero et al. [96]	> 2.0	< − 2.0

Among studies including speed-based intensity zones (*n* = 25), eight studies [45, 78–84] utilized five intensity zones with mean thresholds of 0.0–4.6 ± 1.8 km × h^−1^ (range 2.1–6.0 km × h^−1^), 4.7 ± 1.8 km × h^−1^ (range 2.1–6.0 km × h^−1^) to 9.4 ± 3.4 km × h^−1^ (range 4.9–12.0 km × h^−1^), 9.5 ± 3.4 km × h^−1^ (range 4.9–12.1 km × h^−1^) to 14.5 ± 4.5 km × h^−1^ (range 8.7–18.0 km × h^−1^), 14.6 ± 4.6 km × h^−1^ (range 8.7–18.1 km × h^−1^) to 19.7 ± 5.6 km × h^−1^ (range 12.6–24.0 km × h^−1^), and > 19.7 ± 5.6 km × h^−1^ (range 12.6 to > 24.1 km × h^−1^) across studies. In turn, three studies [39, 40, 85] utilized four speed-based intensity zones with identical thresholds adopted, including < 7.0 km × h^−1^, 7.01–14.0 km × h^−1^, 14.01–18.0 km × h^−1^, and > 18.01 km × h^−1^. Moreover, two studies [86, 87] used three speed-based intensity zones with different zones and thresholds adopted. A further three studies [43, 88, 89] utilized two speed-based intensity zones; however, only movements at higher intensities (> 14.0 km × h^−1^ [88, 89] or > 18.0 km × h^−1^ [43]) were captured. In this regard, the first zone had mean thresholds of 15.3 ± 2.3 km × h^−1^ (range 14.0–18.0 km × h^−1^) to 21.0 ± 0.0 km × h^−1^, while the second zone had a mean initial threshold of > 21.0 ± 0.0 km × h^−1^, with only one study including an upper threshold of 31 km × h^−1^ [89]. Lastly, nine studies utilized one intensity zone to categorize only high-intensity movements, with only a mean initial threshold of > 17.4 ± 1.2 km × h^−1^ (range > 14.4 to > 18.0 km × h^−1^) across studies.

Across studies reporting intensity zones for accelerations and decelerations (*n* = 22), 18 studies [39–42, 44, 80, 82–84, 86, 87, 90–96] only measured high-intensity accelerations and decelerations with an initial mean threshold of > 2.0 ± 0.6 m·s^−2^ (range > 0 to > 3.0 m·s^−2^) across studies. A further two studies [43, 78] utilized two intensity zones for accelerations and decelerations, with one of them including thresholds of < 2.0 m·s^−2^ and > 2.0 m·s^−2^ for accelerations and <  − 2.0 m·s^−2^ and >  − 2.0 m·s^−2^ for decelerations, and the other using thresholds of > 2.0 m·s^−2^ and > 3.0 m·s^−2^ for accelerations and decelerations. Finally, one study utilized five intensity zones to delineate acceleration and deceleration intensities [45], while another study utilized three intensity zones to delineate acceleration and deceleration intensities [97].

## Discussion

Our review is the first to systematically examine variations in the intensity zones and associated thresholds to demarcate zones when using the predominant external load monitoring approaches (i.e., video-based TMA, microsensors, and LPS) utilized in existing basketball research. Our results demonstrate systemic variations exist in the number of zones utilized and the thresholds adopted for many intensity zones within and between monitoring approaches, which limits the ability to make meaningful comparisons across studies or draw definitive conclusions across the collective research published on this topic. Variations in the intensity zones adopted and associated thresholds used to demarcate intensity zones were bound to occur given that no guidelines have been developed to provide researchers with best-practice recommendations. Accordingly, our review emphasizes a clear need for guidance when monitoring and reporting external load variables using intensity zones in future basketball research.

### Video-Based Time-Motion Analysis: Non-numeric Thresholds to Identify Intensity Zones

Variation was evident in the intensity zones adopted across studies using video-based TMA with non-numeric thresholds. More precisely, for locomotion-based movements, most studies (14 out of 18, 78%) included standing, walking, jogging, running, and sprinting, demonstrating these movements are recognized as fundamental in basketball. However, variation predominantly emerged for locomotion-based movements among studies, with standing and walking either included as separate zones (four out of 14 studies, 29%) or combined in the same zone (10 out of 14 studies, 71%). Given that standing and walking are both low-intensity movements that elicit minimal energy expenditure in players compared with running [98], it may be argued that separating them creates additional data to handle and interpret without any further meaningful insight into the external loads experienced. Consequently, including standing and walking in the same intensity zone may reduce the data burden on end-users, allowing for increased focus on movements that evoke greater stress in players.

Wider variation in intensity zones was evident for basketball-specific movements than for locomotion-based movements across TMA studies using non-numeric thresholds. In this regard, although shuffling movements were mostly categorized into low-, moderate-, and high-intensity zones, they were identified differently across studies. Specifically, shuffling movements were identified as shuffling actions of the feet in sideways or backwards directions (six out of 18 studies, 33%) or grouped with various other multi-directional activities in an inconsistent manner as specific movements (seven out of 18 studies, 39%). Consequently, the collective literature indicates shuffling activity is essential to consider, but it appears movements such as backwards and sideways running have been categorized as specific movements alongside shuffling, or grouped collectively with forwards running as locomotion-based movements (i.e., walking, jogging, running, or sprinting). Given that movement direction impacts the external load encountered during locomotor tasks [98–100], and different training approaches are needed to improve backwards, sideways, and forwards running abilities, movements should likely be measured specific to the direction in which they are performed (i.e., backwards, sideways, and forwards) in research for greatest specificity in the reported data. Identification of basketball-specific movements becomes more inconsistent across the literature as some studies included picking/screening (four out of 18, 22%) and positioning (three out of 18, 17%) as separate basketball-specific movement zones, while other studies grouped picking/screening and positioning movements within broader specific zones (five out of 18, 28%). Furthermore, some studies (13 out of 18, 72%) did not quantify picking, screening, or positioning movements, meaning they were likely absorbed in standing, walking, or basketball-specific activity, resulting in underreported player demands. While there are variations across studies regarding the inclusion of picking/screening and positioning, it is likely important to quantify and identify different intensity descriptors for these movements to understand the complete external loads experienced during basketball training and games, given that each of these actions involve extensive player-to-player contact and isometric muscular contractions, which add to the demands experienced [101, 102]. In contrast, jumping movement zones were included in almost all studies (17 out of 18, 94%), emphasizing the widespread recognition of jumping as a fundamental movement to consider when quantifying external load in basketball. Indeed, basketball players execute jumping maneuvers each minute during gameplay [1], which is among the most frequent across a wide range of team sports [103]. The almost unanimous inclusion of jumping as a movement zone may be attributed to the ease of identifying this movement using video-based TMA.

Despite the inconsistencies in categorization of movement zones across studies using video-based TMA with non-numeric thresholds, 14 out of 18 studies (78%) cited the first study published on this topic by McInnes et al. [104]. However, McInnes et al. did not provide any rationale for the approach adopted, making it difficult to identify the underlying logic, processes, and evidence involved in developing their movement zones. Moreover, many authors [105–117], who since cited McInnes et al. [104] in support of their approach, modified the original movement zones, particularly for basketball-specific movements. Modifications to the methods used by McInnes et al. [104] across time may reflect them as being somewhat outdated, as they were published almost three decades ago. Consequently, adaptations to the original methods proposed by McInnes et al. [104] without accompanying justification have likely contributed to inconsistent movement zones being used across studies, emphasizing the need for guidance in establishing fundamental movements that should be considered when quantifying external load with video-based TMA in modern basketball.

### Video-Based Time-Motion Analysis: Numeric Thresholds to Identify Intensity Zones

The included studies utilizing video-based TMA with numeric thresholds were published by four different author groups, with each research group adopting different intensity zones in their research. In this regard, while three of the four author groups (five out of six studies, 83%) identified standing, walking, jogging, running, and sprinting as key locomotion-based movement intensity zones, variations emerged across studies in other ways. Specifically, two author groups separated standing and walking [31, 53], whereas other author groups combined standing and walking in the same zone [54–56] or broadly combined movements as low-, medium-, or high-intensity activity [52]. Moreover, the thresholds used to demarcate locomotion-based movement intensity zones differed across author groups. For instance, jogging was detected using initial thresholds with almost a twofold difference (i.e., from 1.1 m·s^−1^ to 2.1 m·s^−1^), while sprints were detected using thresholds from 6.1 m·s^−1^ to 7.0 m·s^−1^. Moreover, only two author groups (four out of six studies, 67%) included basketball-specific movements [31, 54–56]. Although shuffling activity was included by both author groups that included basketball-specific movements [31, 54–56], it was inconsistently separated into three (low, moderate, and high) [31] or two (low and high) [54–56] intensity zones, with different thresholds adopted between groups. Furthermore, jumping was included as a movement zone by both author groups that included basketball-specific movements, but dribbling [54–56], upper-body movements [54, 56], and sideways running [31] were each included by only one author group.

The extensive variation in the intensity zones included across studies using video-based TMA with numeric thresholds likely stems from the lack of consensus foundation research regarding best practices for using external load intensity zones in basketball research. In this way, most studies adapted key movement zones contained in the original video-based TMA study using non-numeric thresholds by McInnes et al. [104], and then applied different approaches to assign numeric thresholds for these zones. Specifically, some studies adopted numeric thresholds for intensity zones developed in research examining other court-based (i.e., futsal) [52, 54, 56, 118] or field-based team sports (i.e., soccer) [53, 119], diminishing the specificity to basketball, while other studies did not include any reasoning for the numeric thresholds assigned [31, 104]. Consequently, although limited studies have included intensity zones using video-based TMA with numeric thresholds to delineate intensity zones, there is still a need for consistency in approaches, given that automated video analyses may still be used in practice and other newer monitoring approaches utilize similar speed-based zone thresholds.

### Microsensors

The studies utilizing microsensors in this review demonstrated relatively consistent approaches to delineating intensity zones, given that many adopted a single threshold to capture only high-intensity movements (13 out of 33 studies, 39%). More precisely, among studies including only high-intensity zones, most studies used thresholds of > 3.5 m·s^−2^ for accelerations (nine out of 11 studies, 82%), decelerations (nine out of 10 studies, 90%), and COD (five out of five studies, 100%), and used a threshold of > 40 cm for jumps (10 out of 10 studies, 100%). Likewise, consistent thresholds were used across the three studies [58–60] utilizing three intensity zones for accelerations, decelerations, and COD (1.5–2.5 m·s^−2^, 2.5–3.5 m·s^−2^, and > 3.5 m·s^−2^, respectively) as well as for jumps (0–20 cm, 20–40 cm, and > 40 cm, respectively). Despite these similarities in the intensity zones adopted across studies, variations were introduced in the measurement units used between studies. Specifically, five studies measured accelerations using intensity zones defined in g-forces [64–68], while the other 15 studies [15, 32–38, 57–63] utilized m·s^−2^. Furthermore, among the five studies that measured accelerations using intensity zones defined in g-forces, three studies only recorded accelerations > 4 g-forces [66–68], while the other two studies only recorded accelerations up to 2 g-forces [64, 65]. In turn, only reporting accelerations < 2 g-forces [64, 65] and > 4 g-forces [66–68] separately across studies introduces notable variation in the acceleration data reported and may omit important accelerative demands at high or low intensities. Given the five studies that reported accelerations using g-forces were all conducted in basketball players of similar ages (mean age of 13.7–20.0 years) and playing levels (club, college/university, and representative), who were therefore likely to experience similar g-forces during accelerations, the variation in intensity zones adopted by researchers may be attributed to the differing hardware, software, and data processing procedures used across studies, as well as differing justifications for the intensity zones selected. In regard to the differing justification, one study [66] cited badminton and rugby union as the source for the initial intensity zones selected. Additionally, in one study by Bredt et al. [65], the authors stated that selected intensity zones were not based on any prior recommendations or research. The authors' rationale for not utilizing previously published research was because they did not find any existing recommendations for intensity zone classification in basketball. Subsequently, in a second study [64] by Bredt et al., the authors cited their previous paper [65] as justification for the intensity zones despite not providing any rationale for them. The other two studies [67, 68] did not provide any justification. Specifically regarding the hardware and software used, research has noted that discrepancies in microsensor outputs may occur due to variability in software versions, sampling rates, data filtering and smoothing techniques [29], firmware updates [29], and minimum effort durations chosen for detecting movements [120]. Consequently, detailed information encompassing the hardware and software, as well as data sampling, cleaning, and detection processes, should be provided in studies when microsensors and other monitoring approaches such as video-based TMA and LPS are used to measure external load variables in basketball players.

Limited studies (three out of 26, 12%) included intensity zones for PlayerLoad, which is one of the most widely used variables to quantify external load in basketball research [50, 121]. Although these three studies followed manufacturer settings in assigning PlayerLoad intensity zones, it appears that the understanding and acceptance of universal intensity zones are yet to be established for PlayerLoad in basketball contexts across the literature. This may be due to researchers perceiving greater practical utility in quantifying high-intensity accelerations and decelerations, researchers limiting the volume of variables reported, or the arbitrary nature of the unit used to quantify this variable. Nevertheless, all studies [60, 69, 70] that reported intensity zones for PlayerLoad were quantified in AU per minute and consistently applied across five [69] or six fixed intensity zones [60, 70]. However, PlayerLoad intensity zone thresholds were also quantified using relative intensity zones (in addition to fixed intensity zones) that were calculated relative to the individualized peak PlayerLoad achieved in AU per min during training or games in a study [70], which has also been applied in other court-based team sports [122]. Likewise, the seven studies [71–77] measuring average net force with microsensors included in our review also delineated intensity zones using relative thresholds stratified using different percentages of VO_2_R, which were calculated on the basis of individualized associations between the average net force and average VO_2_R across different stages of a modified Yo–Yo Intermittent Recovery Test. Surprisingly, these eight studies [70–77] were the only instances in which relative intensity zone thresholds were adopted in our review, with most studies (*n* = 78) utilizing fixed intensity zones thresholds.

Use of intensity zone thresholds relative to peak outputs attained during training sessions and games [123, 124], maximal speed attained during linear sprint assessments [125, 126], and intensity markers such as speed at lactate [127] and ventilatory thresholds [128] attained during physiological fitness assessments have been widely adopted for monitoring approaches in field-based team sports. Although fixed intensity zone thresholds may permit players to be benchmarked against desired criteria or one another, and involve simpler processes during analyses in software for end-users [20], use of relative intensity zone thresholds accounts for variations in fitness status [129] and performance capacities [123, 125, 130] across players to provide external load data that may allow for greater individualization in developing training plans and player recovery strategies [128]. However, the added practical requirement to continually measure and adjust individualized thresholds due to longitudinal changes in fitness capacities among players when using relative intensity zones needs to be considered [131]. Likewise, the translation of relative intensity zone thresholds derived from continuous running assessments to competitive gameplay has been questioned given the extensive COD and accelerative requirements in team sports [19], particularly in basketball [103]. Consequently, there appears to be logical utility in using both relative and absolute intensity zone thresholds in basketball dependent on the intended application, with considerably more research needed to identify optimal approaches.

The greater consistency in intensity zone thresholds across studies using microsensors compared with studies using video-based TMA likely stems from a clear reliance on the use of manufacturer settings. Most studies (22 out of 26, 85%) used Catapult devices to capture external load data, with the included intensity zone thresholds being rather consistent given they align directly with those specified within predefined manufacturer settings. This reliance is somewhat concerning given that the rationale and evidence used to define the intensity thresholds set by manufacturers remain undisclosed, creating a need for greater transparency alongside further independent research and expert input to understand whether manufacturer-derived intensity zones are appropriate to implement in practice.

### Local Positioning Systems

Similar to studies utilizing video-based TMA with numeric thresholds, variation in speed-based intensity zone thresholds among LPS studies (*n* = 25) were mostly evident across lower intensity zones. For instance, considerable variance in intensity thresholds was apparent between studies, including low-intensity zones, with the initial thresholds ranging from < 2.14 km × h^−1^ to < 7 km × h^−1^ across studies. This variation may be due to two studies including standing as a separate zone [45, 79], with eight studies combining standing and walking in the same zone and two studies not referring to “standing” activity at all [78, 86]. These findings emphasize the notion that it may be preferable to combine standing and walking activity rather than consider them as separate zones. Considering higher intensity zones, several studies (12 out of 25, 48%) only reported movements above 14 km × h^−1^. Moreover, most studies (16 out of 20, 80%) that specifically referred to “high-speed running” or “high-intensity running” utilized relatively consistent thresholds of ~ 18 km × h^−1^ on its own or with an upper limit of 24 km × h^−1^ (Table 8). Likewise, for sprinting activity, most studies (seven out of 12, 58%) utilized a threshold of > 24.1 km × h^−1^, with some (three out of 12, 25%) adopting a threshold of > 21 km × h^−1^. Regarding the use of LPS encompassing acceleration and deceleration intensity zones, most studies (14 out of 22, 64%) utilized a single intensity zone threshold of > 2 m·s^−2^, with four studies (18%) using an alternative threshold for the single zone (ranging from 0–3 m·s^−2^) and four studies (18%) including multiple intensity zones (ranging from 2 to 5 zones).

The high consistency in approaches to demarcating intensity zones across studies may be attributed to the collective LPS research being predominantly published by six author groups (21 out of 25 studies for speed-based intensity zones, 84%; 21 out of 22 for acceleration and deceleration intensity zones, 98%) who used either Catapult ClearSky or WIMU PRO monitoring systems. Importantly, no studies utilized > 3.5 m·s^−2^ as a threshold for high-intensity accelerations and decelerations, which was consistently utilized across the microsensor studies included in our review. This discrepancy raises a clear concern when attempting to compare reported acceleration and deceleration data between studies using microsensors and LPS to collect data. Nevertheless, the collective findings across microsensor and LPS studies indicate researchers are mostly interested in quantifying high-intensity accelerations and decelerations during training and games, potentially due to the high biomechanical demands they elicit [132].

Of note, one study [45] developed specific intensity zone thresholds using LPS for different playing levels (i.e., first division versus second division of Liga Femenina) to account for the varied external loads players experience. More precisely, the authors used monitoring data collected during training scrimmage scenarios and k-means clustering to develop intensity zones for speed-based and acceleration/deceleration variables for each playing level, which were all higher in the first division group [45]. Given the variations in external game loads reported between playing levels [101, 105, 112, 133], as well as between competitions involving different sexes [55] and age groups [87], such approaches may hold merit for developing intensity zone thresholds in basketball research to better categorize acquired data. Similarly, it has been suggested that universal fixed intensity zone thresholds of equal bandwidth may be used, but the corresponding label assigned to each zone (e.g., jog, run, sprint) could be adjusted dependent upon the playing level, sex, and/or age of the sample to account for differences in external loading [131], or expanding on this idea by using consistent fixed zones without labels. A further noteworthy observation across LPS studies was thresholds for acceleration and deceleration data were inconsistently, and often inaccurately, reported using positive (+) or negative (−) values, as well as less-than (<) or greater-than (>) signs. Although it appears that authors used similar intensity zone threshold values across studies, it is recommended that deceleration data be identified using negative values and less-than signs to indicate increased deceleration intensity for correctness.

### Limitations

While our review is the first to systematically identify the external load intensity zones and thresholds adopted in basketball literature using popular monitoring approaches, some limitations should be acknowledged. Firstly, syntheses of intensity zone thresholds specific to the sex, age, and playing level of players for each monitoring approach could not be made due to the limited scope of player samples recruited in studies. For instance, limited studies exclusively examined female players (19 of 86, 22%), most studies examined player samples with mean ages between 18 and 27 years (51 out of 86, 59%), and most studies examined athletes competing at the semi-professional or professional level (53 out of 86, 62%). Secondly, optical tracking systems were not included as a predominant external load monitoring approach given they are an emerging technology with limited uptake in basketball research to date. As basketball studies using optical tracking systems become more prevalent, any recommendations developed concerning the development of external load intensity zones and thresholds should encompass this technology. Thirdly, the provided outcomes are specific to basketball and should not be extrapolated to other court-based sports such as netball [134], handball [103], or wheelchair basketball [135] given their different external demands.

### Future Research Directions

The overarching future recommendation stemming from our review is the need to develop consensus guidelines that promote more sound and consistent approaches when monitoring external load variables using intensity zones. In this regard, an initial step in creating such guidelines could involve a Delphi study to elucidate current consensus on developing external load intensity zones and intensity thresholds among experts working within this field [136]. Validation studies are then needed to support the application of consensus approaches in setting external load intensity zones and thresholds for their intended practical functions. For instance, studies could explore the associations between external load data derived using suitable fixed and relative intensity zone thresholds for various practical functions, such as quantifying changes in fitness attributes, injury incidence, and in-game performance among different player samples [137]. Regarding relative intensity zone thresholds, various methods to set zone thresholds should be explored to identify the most effective approaches that are also practical to implement in basketball teams given the apparent lack of basketball research using them to date. Moreover, the reliance on manufacturer settings to set intensity zone thresholds using microsensors and LPS across the literature emphasizes the important role that manufacturers play in determining reported external load data and the need to ensure that transparent, consistent, evidence-informed approaches are adopted across different software platforms. A stronger evidence base established from practitioner expertise and scientific data could potentially inform the development of a consensus statement that guides researchers and practitioners in appropriately setting external load intensity zones. Such guidance could entail minimum reporting standards in describing and justifying the adopted methods as well as in providing appropriate absolute and relative intensity zones and thresholds for application in specific player samples considering their age, sex, and playing level when monitoring external load with different technologies.

## Conclusions

This is the first review to examine the approaches adopted to classify external load intensity zones and thresholds in basketball research, showing that systemic variations exist across studies using the same or different (i.e., microsensors versus LPS or video-based TMA versus LPS) external load variable monitoring approaches. Although research using video-based TMA with non-numeric and numeric thresholds somewhat consistently accounted for jogging, running, sprinting, and jumping movements, some notable inconsistencies were evident in the approaches adopted. Namely, studies inconsistently combined or separated standing and walking activity, included varied basketball-specific movements, and did not uniformly or comprehensively account for the range of multi-directional and isometric activities performed. In research using microsensors, many studies included zones for accelerations, decelerations, COD, and jumps only performed at high intensities with relatively consistent thresholds adopted that aligned with manufacturer software settings. Surprisingly, despite the popularity of using PlayerLoad to measure total external load volume in the basketball literature, limited research has demarcated PlayerLoad into intensity zones when quantifying external load with microsensors. Like microsensor research, studies using LPS predominantly included zones only encompassing high-intensity movements but in a consistent manner and in line with manufacturer software settings. However, the intensity zone thresholds set to detect accelerations and decelerations using LPS (2–3 m·s^−2^) were notably lower than those adopted in studies using microsensors (3.5 m·s^−2^), highlighting discrepancies between monitoring approaches. Of note, limited research has explored the use of relative intensity zone thresholds when quantifying external load variables in basketball research, emphasizing an important area in need of further research attention given the popularity of using relative intensity zones in wider team sports [19–22]. The inconsistencies in the number and type of intensity zones utilized, as well as the associated thresholds for a given intensity zone among basketball studies using popular monitoring approaches (i.e., video-based TMA, microsensors, and LPS) support the need for further research to inform the development of consensus guidelines that outline best-practice approaches when implementing external load intensity zones and intensity thresholds in research and practice.

## Declarations

### Availability of data and materials

Data and calculations for mean ± standard deviation intensity zones and thresholds reported in our review can be provided upon request.
